# An examination of health care utilization during the COVID-19 pandemic among women with early-stage hormone receptor-positive breast cancer

**DOI:** 10.1186/s12913-022-08705-9

**Published:** 2022-11-23

**Authors:** Andrew J. Paladino, Kinsey Pebley, Mehmet Kocak, Rebecca A. Krukowski, Teresa M. Waters, Gregory Vidal, Lee S. Schwartzberg, Andrea N. Curry, Ilana Graetz

**Affiliations:** 1 College of Medicine, The University of Tennessee Health Science Center, Memphis, TN USA; 2 Department of Psychology, University of Memphis, Memphis, TN USA; 3 International School of Medicine, Biostatistics and Medical Informatics, Istanbul Medipol University, Uskudar, Istanbul, Turkey; 4 Department of Public Health Sciences, University of Virginia, University of Virginia Cancer Center, Charlottesville, VA USA; 5 Department of Health Management and Policy, University of Kentucky College of Public Health, Lexington, KY USA; 6 West Cancer Center and Research Institute, Department of Medical Oncology, Germantown, TN USA; 7 Department of Health Policy and Management, Emory University Rollins School of Public Health, Atlanta, GA USA; 8 Medical Oncology and Hematology, Renown Institute for Cancer, Reno, USA

**Keywords:** COVID-19, Breast Cancer, Coronavirus pandemic, Health care utilization

## Abstract

**Background:**

Women undergoing treatment for breast cancer require frequent clinic visits for maintenance of therapy. With COVID-19 causing health care disruptions, it is important to learn about how this population’s access to health care has changed. This study compares self-reported health care utilization and changes in factors related to health care access among women treated at a cancer center in the mid-South US before and during the pandemic.

**Methods:**

Participants (*N* = 306) part of a longitudinal study to improve adjuvant endocrine therapy (AET) adherence completed pre-intervention baseline surveys about their health care utilization prior to AET initiation. Questions about the impact of COVID-19 were added after the pandemic started assessing financial loss and factors related to care. Participants were categorized into three time periods based on the survey completion date: (1) pre-COVID (December 2018 to March 2020), (2) early COVID (April 2020 – December 2020), and later COVID (January 2021 to June 2021). Negative binomial regression analyses used to compare health care utilization at different phases of the pandemic controlling for patient characteristics.

**Results:**

Adjusted analyses indicated office visits declined from pre-COVID, with an adjusted average of 17.7 visits, to 12.1 visits during the early COVID period (*p* = 0.01) and 9.9 visits during the later COVID period (*p* < 0.01). Hospitalizations declined from an adjusted average 0.45 admissions during early COVID to 0.21 during later COVID, after vaccines became available (*p* = 0.05). Among COVID period participants, the proportion reporting changes/gaps in health insurance coverage increased from 9.5% participants during early-COVID to 14.8% in the later-COVID period (p = 0.05). The proportion reporting financial loss due to the pandemic was similar during both COVID periods (34.3% early- and 37.7% later-COVID, p = 0.72). The proportion of participants reporting delaying care or refilling prescriptions decreased from 15.2% in early-COVID to 4.9% in the later-COVID period (p = 0.04).

**Conclusion:**

COVID-19 caused disruptions to routine health care for women with breast cancer. Patients reported having fewer office visits at the start of the pandemic that continued to decrease even after vaccines were available. Fewer patients reported delaying in-person care as the pandemic progressed.

**Supplementary Information:**

The online version contains supplementary material available at 10.1186/s12913-022-08705-9.

## Introduction

Women with early stage, hormone-receptor positive (HR+) breast cancer who are undergoing primary treatments (i.e., surgery, radiation, chemotherapy) often experience many physical (e.g., pain, lymphedema, nausea and vomiting) [1–6] and psychological side effects (e.g., depressive symptoms, body image distress, anxiety) [7, 8]. Thus, close monitoring and frequent follow-up is important for maintenance of treatments, curative outcomes, and prevention of cancer recurrence [9–12]. After completing primary treatment, most will be prescribed adjuvant endocrine therapy (AET) for many years to help prevent recurrence. Regular visits and open commutation are crucial to optimize the cancer care environment, foster long-term treatment adherence, and improve health outcomes [13, 14].

The COVID-19 pandemic has disrupted health care delivery by discouraging in-person use of services to curtail the spread of the virus. The effect of the COVID-19 pandemic and the advent of the vaccine on health care utilization among breast cancer patients are not yet fully understood. One study examined older breast cancer survivors and found that they reported significant disruptions to health care within the first six months of the pandemic, but the magnitude of these disruptions has not yet been elucidated [15]. Another study showed a 18% decrease in treatment volume in 2020 compared to 2018 for breast cancer at a large academic medical center; however, they noted no delays from diagnosis to initiating adjuvant therapy [16]. Thus, it is critically important to assess how this high-risk population’s access to and utilization of health care has changed since the pandemic began and since vaccinations became available to adapt care delivery options and optimize health outcomes.

The pandemic has had myriad other documented effects on various aspects of breast cancer care, including decreased mammography screening [17, 18], changes to treatment protocols [19], and greater delays and disruptions in radiotherapy and surgeries during primary treatment [20]. To our knowledge, no prior studies have described the impact of the COVID-19 pandemic on health care utilization behaviors or barriers to health care access among women with early-stage, HR + breast cancer within the 6-month period prior to initiating the adjuvant phase of treatment. Prior research indicates that from the point of breast cancer diagnosis to initiating AET on average takes approximately 9 months [21], so examining the 6-month period prior to starting AET is a useful estimation of the primary phase of breast cancer care. Prior research has focused on delayed care visits and decreased contact with oncology providers among women who were up to 5 years after diagnosis and among women presenting for a surgical consultation and did not distinguish between different periods of the pandemic [22, 23]. The current study aimed to address this gap by examining health care access and utilization over the prior 6 months among women with early-stage, HR + breast cancer newly prescribed AET in pre-COVID, early COVID (pre-vaccination), and later COVID (post-vaccination) periods.

## Methods

### Study Design

Between December 2018 and June 2021, women with early-stage, HR + breast cancer newly prescribed AET at a large integrated outpatient cancer facility in the mid-South US were invited to participate in a longitudinal study that aimed to improve adherence to AET. Like health care providers across the country, the cancer network quickly pivoted to provide the option of telehealth, including video and telephone visits, in March of 2020. Notably, the center never closed to in-person visits, but providers were encouraged to move appointments to telehealth when possible to mitigate the spread of COVID-19.

All participants completed a baseline survey upon enrollment, which included assessments of demographics, disease characteristics, and health care utilization within the last six months. Demographic data included age, gender, race, income level, marital status, and education. Disease characteristics included disease stage and type of AET used. Health care utilization questions captured self-reported number of office visits, urgent care/emergency department visits, and hospital admissions over the prior six months.

The original study design has been detailed previously [24]; however, alterations were made, and additional measures were added to assess the impact of the COVID-19 pandemic. New questions related to health care utilization during the pandemic were added in March 2020. Participants recruited after March 2020 completed additional questions assessing the number of telephone and video visits, and the impact of COVID-19 on their financial situation, insurance coverage, and delays in in-person care. These questions asked participants to indicate to what extent (“not at all,” “slightly,” “moderately,” “considerable,” “a great deal”) they experienced personal financial loss, changes or gaps in health insurance, or delayed in-person care or prescription refills. Moreover, we revised our recruitment effort to be completed fully remotely, without requiring an in-person visit after March 2020. Informed consent was obtained from all study participants. This study has been approved by the organization’s Institutional Review Board.

### Statistical analysis

Participants were categorized into the following three time periods based on the date they completed the enrollment survey: (1) pre-COVID, before the COVID pandemic precautions began (December 2018 to March 2020), (2) early COVID, prior to vaccine availability (April 2020 – December 2020), and later COVID, following vaccine availability (January 2021 to June 2021). March 6, 2020 was chosen as the date to separate pre-COVID and the pre-vaccination era because this was the date the study paused and changes to study protocols were implemented due to the emergence of COVID and precautions. January 1, 2021 was selected as the date to separate the pre-vaccination and post-vaccination eras because this was when vaccines started to become available in the study region. Participant access to vaccines depended on their situation (e.g., their occupation, age, risk status, etc.), as vaccine accessibility was phased. Responses related to the impact of the pandemic on finances, health insurance, and prescription fills were dichotomized into “any impact” and “no impact.”

Missing data was minimal, less than 2% among outcome variables and less than 6% among the control variables. Specifically, only six participants had missing data related to office visits (2%), and four had missing data for hospital admissions and urgent care/ER visits (1.3%). Listwise deletion was used for missing outcome variables. As sensitivity analyses, we generated 100 samples utilizing the SAS MI procedure to impute missing control variables with fully conditional specification approach. The model results based on these imputed data were practically the same as main results in Table 2 (Supplementary Table 1).

Descriptive statistics were calculated for demographic and COVID-19 impact variables (i.e., financial loss, insurance changes, delays in care). Continuous variables capturing the frequency of health care utilization (i.e., office visits, urgent care/ER visits, hospital admissions, and telephone/video visits) were compared among the three time periods. Continuous variables were compared among COVID-19 time periods using Wilcoxon-Mann-Whitney or Kruskal-Wallis tests.

Vuong and Clarke tests [25, 26] indicated that, compared to zero-inflated Poisson, Negative Binomial Regression (NBR) models provided better fit for the health care utilization outcomes. Therefore, multivariable models were constructed as NBR models with health care utilization counts as the outcome variable, COVID-19 time periods as the primary predictor, controlling for age, education, poverty status, marital status, and disease stage. Additional sensitivity analyses conducted using different washout periods (30 to 180 days) in between pre- and early-COVID to minimize overlap between pre- and COVID periods and limiting the early-COVID period to six months. Results from these analyses were consistent in direction and significance as the main results (Supplementary Tables 2A, 2B, and 2C). A type-1 error rate of 0.05 was considered as the significance threshold.

## Results

### Participant characteristics

Overall, 306 women completed the survey (N = 140 pre-COVID, N = 57 early-COVID, and N = 109 later-COVID). Participants were mostly from White (63.7%) or Black (32.7%) racial identities, 50.7% had less than a 4-year college degree, and most were living with a partner and/or were married (66.0%). Over half of participants reported incomes higher than 400% of the federal poverty line (FPL), while 10.5% had incomes below 100% FPL. Most participants (75.5%) were prescribed an aromatase inhibitor (Anastrozole, Exemestane, or Letrozole) and 24.6% Tamoxifen. Participant characteristics were not statistically significantly different between the three time periods. Table 1 displays participant characteristics for each period.

**Table 1:** Respondent Characteristics by Study Period

Characteristic	Pre-COVID (*n* = 140)	Early-COVID (*n* = 57)	Later-COVID (*n* = 109)	Total Sample (*N* = 306)	
	***n*** **(%)**	***n*** **(%)**	***n*** **(%)**	***N*** **(%)**	***p***
**Race**					0.38
Black	41 (29.3%)	34 (32.4%)	25 (41.0%)	100 (32.7%)	
White	93 (66.4%)	67 (63.8%)	35 (57.4%)	195 (63.7%)	
Other	3 (2.1%)	4 (3.8%)	1 (1.6%)	8 (2.6%)	
Missing	3 (2.1%)	0 (0.0%)	0 (0.0%)	3 (1.0%)	
**Education**					0.57
< 4-year college degree	67 (47.9%)	52 (49.5%)	36 (59.0%)	155 (50.7%)	
4-year college degree or more	73 (52.1%)	52 (49.5%)	25 (41.0%)	150 (49.0%)	
Missing	0 (0.0%)	1 (0.9%)	0 (0.0%)	1 (0.3%)	
**Income Level**					0.48
Below poverty line	14 (10.0%)	12 (11.4%)	6 (9.8%)	32 (10.5%)	
100-200% above poverty line	13 (9.3%)	13 (12.4%)	7 (11.5%)	33 (10.8%)	
201–400% above poverty line	29 (20.7%)	20 (19.1%)	17 (27.9%)	66 (21.6%)	
> 400% above poverty line	80 (57.1%)	54 (51.4%)	30 (49.2%)	164 (53.6%)	
Missing	4 (2.9%)	6 (5.7%)	1 (1.6%)	11 (3.6%)	
**Marital Status**					0.99
Living with partner/married	91 (65.0%)	66 (62.9%)	45 (73.8%)	202 (66.0%)	
Missing	0 (0.0%)	1 (0.9%)	0 (0.0%)	1 (0.3%)	
**Disease Stage**					0.68
I/IA/IB	92 (65.7%)	72 (68.6%)	47 (77.1%)	211 (68.9%)	
II/IIA/IIB/IIIA/Others	41 (29.3%)	32 (30.5%)	14 (22.9%)	87 (28.4%)	
Missing	7 (5.0%)	1 (0.9%)	0 (0.0%)	8 (2.6%)	
**Age –** ***M*** **(** ***SD*** **)**	59.44 (10.1)	57.62 (12.0)	58.69 (10.5)	58.66 (10.9)	0.55

***Note.*** M = mean; SD = standard deviation. P-values compare characteristics by study period

### Changes in Health care accessibility

**Fig. 1:**
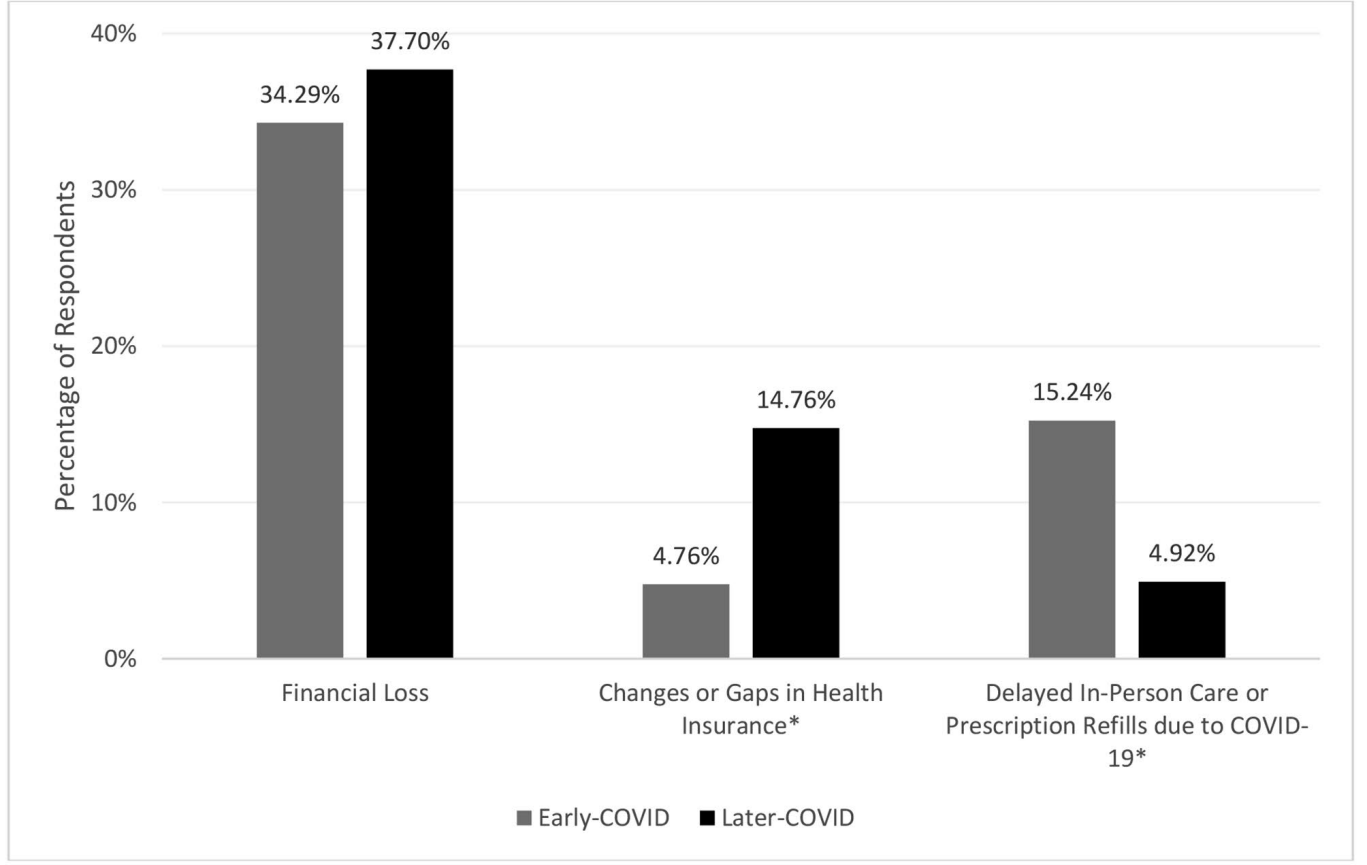
COVID-19 impact on financial loss, insurance coverage, and care delays in early versus later-COVID periods *Note.* *Indicates statistically significant difference between early-COVID and later-COVID (*p* < 0.05) in both the unadjusted and adjusted models controlling for age, education status, below-poverty status, marital status, and disease stage. Data related to these variables were only collected after the COVID pandemic started, and thus comparisons to the pre-COVID period were not possible.

Figure 1 displays changes in factors related to health care access due to COVID-19 between the post-COVID study periods. The proportion reporting changes/gaps in health insurance coverage increased from 9.5% participants during early-COVID to 14.8% in the later-COVID period (*p* = 0.047). The proportion reporting financial loss due to the pandemic was similar during both COVID periods (34.3% early- and 37.7% later-COVID, *p* = 0.72). The proportion of participants reporting delaying care or refilling prescriptions decreased from 15.2% in early-COVID to 4.9% in the later-COVID period (*p* = 0.04).

### Health care utilization

**Fig. 2:**
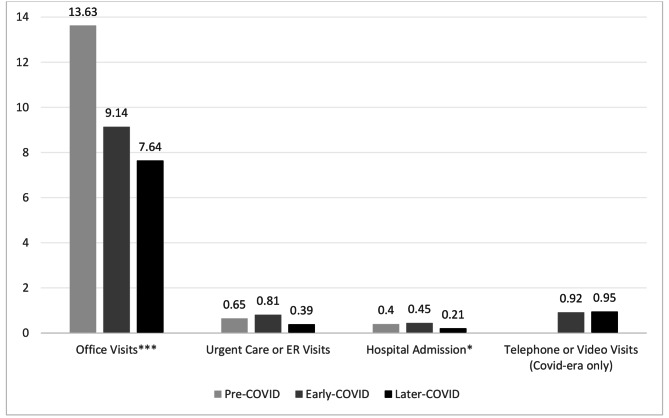
Health care Utilization Means *Note.* Results are from adjusted binomial negative regression model assessing for differences in health care utilization between each period. Data related to telephone and video visits were only collected after the COVID pandemic started, and thus comparisons to the pre-COVID period were not possible. *p < 0.05; ***p < 0.001

Figure 2 shows the adjusted means for reported health care utilization in the prior six months by study period, and Table 2 shows the health care utilization event ratios between each period. The unadjusted Kruskal-Wallis tests showed that office visits were significantly different between time periods (*p* < 0.001). The adjusted negative binomial regression model showed a 32% reduction in office visits from pre-COVID to the early COVID (*p* = 0.010). The adjusted average number of office visits dropped from 17.7 visits during the prior 6 months pre-COVID to 12.1 visits during the early COVID. Office visits declined by 44%, to approximately 9.9 visits, from pre-COVID to the later COVID period (*p* = 0.001). The number of office visits declined 18% from early- to later-COVID periods but did not reach statistical significance (*p* = 0.30).

**Table 2:** Differences in Health care Utilization between Time Periods

Point Estimates and Event Ratios	Event Ratio	95% CI	*p*
**Office Visits**			
Pre-COVID vs. early COVID	0.68	0.50, 0.92	0.01
Pre-COVID vs. later COVID	0.56	0.39, 0.80	< 0.01
Early COVID vs. later COVID	0.82	0.57, 1.19	0.30
**Hospital Admissions**			
Pre-COVID vs. early COVID	1.06	0.65, 1.71	0.83
Pre-COVID vs. later COVID	0.52	0.26, 1.04	0.06
Early COVID vs. later COVID	0.49	0.24, 1.00	0.05
**Urgent Care/Emergency Room Visits**			
Pre-COVID vs. early COVID	1.23	0.60, 2.49	0.57
Pre-COVID vs. later COVID	0.58	0.24, 1.36	0.21
Early COVID vs. later COVID	0.47	0.19, 1.18	0.11
**Telephone and Video Care Visits**			
Early COVID vs. later COVID	0.96	0.55, 1.68	0.89

***Note.*** CI = confidence interval. Models controlled for age, education, poverty status, marital status, and disease stage. Data related to telephone and video visits were only collected after the COVID pandemic started, and thus comparisons to the pre-COVID period were not possible

The unadjusted Kruskal-Wallis tests showed a trend for fewer hospital admissions, from 0.45 admissions over six months during early COVID to 0.21 during later COVID, after vaccines were available (*p* = 0.05). The adjusted models also found 51% fewer hospitalizations in later vs. early COVID (*p* = 0.05). Similarly, the adjusted model found a 53% decline in urgent care/emergency room visits in the later COVID period compared with early COVID, but the change did not reach statistical significance (Table 2, *p* = 0.11). The average number of telehealth visits in the prior six months was similar in early vs. later-COVID periods (0.95 and 0.92, *p* = 0.89).

## Discussion

This study highlights differences in health care utilization by women receiving primary treatment for early-stage hormone receptor-positive breast cancer before the pandemic, during the early pandemic period, before any vaccines were available, and later in the pandemic, after vaccines were available. We found that office visits were significantly higher before the pandemic and continued to decline even after vaccines were available. Over a third of respondents experienced financial loss and nearly one in ten reported having gaps or changes in their health insurance due to the pandemic.

Consistent with prior research suggesting cancer care disruptions and delays [15, 27, 28], our findings demonstrate that, compared to pre-COVID levels, in-person office visits significantly decreased during the pandemic for women with breast cancer in our study. These findings elaborate upon and quantify the disruptions highlighted by Dilwari and colleagues (2021) in the first six months of the COVID pandemic. In contrast to our study, they used a binary “yes/no” response to assess disruptions to medical care, while our study measured the reported number of office visits within before and during the pandemic. Study findings further corroborate the findings of others who showed decreased breast cancer patient volume the year the pandemic began compared to a pre-pandemic level [16]. The lower number of office visits during the pandemic is likely due to mitigation efforts focused on avoiding in-person interactions and potential virus exposures within this medically vulnerable population [29]. In addition, a recent study indicated that most women with breast cancer undergoing surgical treatment expressed anxiety about becoming infected with COVID-19, as well as concerns about the ways that the COVID pandemic might alter their cancer care and recovery [23]. In the same study, approximately two-thirds of study participants utilized telehealth services to reduce their risk of exposure to COVID, however, one-third reported dissatisfaction with their telehealth experience [23].

In addition to individual decisions about seeking care, there were also institutional shifts that drove differences in utilization, including increased surgical and radiotherapy deferrals by physician recommendation [18–20]. Indeed, the cancer network that the study recruited from instituted policies to help mitigate the spread by encouraging oncology providers to move appointments to telehealth whenever possible early in the pandemic. Furthermore, policies external to the institution, such as shelter-in-place policies at the city and state level, precluded continuing in-person visits as usual. Importantly, our findings suggest that use virtual care (telephone or video visits) was low, about one visit over six months, and did not compensate for the steep decline in office visits. The lack of change in office visits to reach closer to “normal” pre-COVID levels even after vaccines became available may reflect continued reluctance for patients to return to office visits. Future studies should evaluate how the decline in in-person visits for patients during the pandemic changed ongoing health care utilization preferences and behaviors, and if these changes alter cancer outcomes.

The economic impact of the pandemic may also have contributed to the continued lower use of office visits, even after vaccines were available and shelter-in-place policies lifted. Over one-third of respondents reported experiencing financial loss due to the pandemic, and the proportion that reported having changes or gaps in their health insurance nearly tripled in the later pandemic period (from 4.8% early COVID to 14.8% later COVID). Similarly, prior studies examining the impact of the pandemic on women with breast cancer noted financial challenges, difficulty obtaining treatment, and dissatisfaction with the lack of information for cancer-specific support during the pandemic [30]. Despite finding continued less frequent office visits in the later-COVID period, the percentage of participants who reported delaying care because of the pandemic decreased substantially in the later COVID period, (from 15.25% in early to 4.92% later COVID). More research is needed to elucidate the reasons for the prolonged decrease in office visits, such as increased use of patient portals or other modalities of communication between providers and patients and changes in insurance or other barriers to care.

For our sample of women receiving primary treatment for early-stage hormone receptor-positive breast cancer, in-person office visits declined by nearly a third at the start of the pandemic and did not recover to pre-COVID levels after vaccines became available. This finding is important given that research suggests that, among women with breast cancer, physical and psychological symptoms worsened during the pandemic [31], and the potential disruptions in care among this group could result in missed recognition of recurrences. It is important for health policy stakeholders and clinicians to understand whether exposure to telehealth during the pandemic change longer-term use of telehealth and in-person visits. Future studies should also examine whether the reductions in frequency of health care utilization persist, and whether these reductions impact treatment adherence and health outcomes. Furthermore, whether there are longer-term and indirect sequelae as a result of the gaps in care from the pandemic, such as damaged patient-provider relationships; missed information that might have facilitated adherence; or continued low screening adherence in the survivorship phase, should be investigated.

## Limitations

Despite its strengths, including the novelty of the research and its exploration of a vulnerable group comprised of a diverse sample of women, this study has some limitations worth mentioning. First, health care utilization data was collected via self-report, did not distinguish between cancer-care visits vs. other health care visits, and was not verified with other sources, such as electronic health records. Similarly, certain survey questions (i.e., changes in health coverage and gaps in insurance, delayed in-person care and delayed prescription refills) were compound questions on the survey, so we were unable to disentangle specific concepts in our analysis. Second, the later COVID, post-vaccination period that we defined was based on when vaccines were approved and distributed using a phased plan in the study region, not necessarily when individuals received them. We contend that health care workers and cancer patients were among the first to be eligible to receive vaccines after emergency approval was given [32], which allowed many of the mitigation strategies to start easing and signaled a more hopeful shift in the perceptions of the public around the pandemic even if they had not received the vaccine yet themselves. Third, there is overlap between the study periods, given that the study examined retrospective 6-month health care utilization, which might possibly bias the comparison between the later-COVID and early-COVID eras. We attempted to account for this by running sensitivity analyses which varied the start and end points of the periods, and the washout-periods between the periods and found consistent results with the main analyses  (Supplementary Tables 2A, 2B, and 2C). Fourth, given that patients were voluntarily enrolled in a trial utilizing communication with their providers, the population of breast cancer patients in our sample might represent one that is more engaged with care than patients who did not choose to participate. Lastly, this study recruited from a single cancer network in the mid-South US and has a small sample size, so findings may not be generalizable to other regions of the country or other cancer centers that incorporated different policies or experienced regional variation in COVID cases.

## Conclusion

COVID-19 caused disruptions to routine health care for women with breast cancer. Women receiving primary treatment for early-stage hormone receptor-positive reported having fewer office visits at the start of the pandemic that continued to decrease even after vaccines were available. Fewer patients reported delaying in-person care as the pandemic progressed. Researchers and policymakers should closely monitor the shifting landscape of cancer care and adapt care delivery in ways that maximize patient safety while striving to provide high quality of care.

## Electronic supplementary material

Below is the link to the electronic supplementary material.

Supplementary Material 1

## Data Availability

The datasets generated during and/or analyzed during the current study are available from the corresponding author on reasonable request.

## List of abbreviations

HR+: Hormone receptor positive
AET: Adjuvant endocrine therapy
NBR: Negative Binomial Regression
