# Supplementary material for: An examination of health care utilization during the COVID-19 pandemic among women with early-stage hormone receptor-positive breast cancer
Source: BMC Health Serv Res. 2022 Nov 23;22:1403. doi: 10.1186/s12913-022-08705-9 (PMC9684812; doi:10.1186/s12913-022-08705-9)
Supplement: Supplementary file 1 — Supplementary Material 1 [file 12913_2022_8705_MOESM1_ESM.docx]

Supplementary Table 1. *Differences in Healthcare Utilization between Time Periods using 100 Multiply Imputed Data (Presented values are the medians over 100 imputed samples)*

| **Point Estimates and Event Ratios** | **Event Ratio** | **95% CI** | ***p*** |
| --- | --- | --- | --- |
| **Office Visits** |  |  |  |
| Pre-COVID vs. early COVID | 0.68 | 0.51, 0.90 | <0.01 |
| Pre-COVID vs. later COVID | 0.54 | 0.38, 0.77 | <0.01 |
| Early COVID vs. later COVID | 0.80 | 0.56, 1.16 | 0.24 |
| **Hospital Admissions** |  |  |  |
| Pre-COVID vs. early COVID | 1.09 | 0.67, 1.76 | 0.97 |
| Pre-COVID vs. later COVID | 0.56 | 0.28, 1.11 | 0.10 |
| Early COVID vs. later COVID | 0.51 | 0.25, 1.04 | 0.07 |
| **Urgent Care/Emergency Room Visits** |  |  |  |
| Pre-COVID vs. early COVID | 1.27 | 0.63, 2.54 | 0.51 |
| Pre-COVID vs. later COVID | 0.60 | 0.26, 1.41 | 0.24 |
| Early COVID vs. later COVID | 0.48 | 0.19, 1.18 | 0.11 |
| **Telephone and Video Care Visits** |  |  |  |
| Early COVID vs. later COVID | 1.03 | 0.59, 1.81 | 0.91 |

*Note.* CI = confidence interval. Adjusted negative binomial regression model controlled for age, education, poverty status, marital status, and disease stage. Data related to telephone and video visits were only collected after the COVID pandemic started, and thus comparison to the pre-COVID period were not possible.

We conducted sensitivity analyses using different washout periods between the pre- and early-COVID periods and using only a 6-month window for early-COVID. Washout periods included the number of days between pre and early-COVID that were excluded from this sensitivity analyses to minimize overlap between pre- and COVID periods. Results were consistent in direction and significance across models.

Supplementary Table 2A shows the included and excluded sample size, start and end date per period using different washout lengths. The pre-COVID period remains unchanged, but the dates for early- and later-COVID change, and their sample became smaller as the washout period increases.

Supplementary Table 2B shows the washout sensitivity analyses results for office visit utilization event ratios between each period using adjusted negative binomial regression model controlling for age, education, poverty status, marital status, and disease stage.

Supplementary Table 2C shows the washout sensitivity analyses results for hospitalization utilization event ratios between each period using adjusted negative binomial regression model controlling for age, education, poverty status, marital status, and disease stage.

**Supplementary Table 2A. Dates and Sample Sizes per Study Period for the Washout Sensitivity Analyses**

|  | N | First date | End date |
| --- | --- | --- | --- |
| **No washout** |  |  |  |
| 1. Pre-COVID | 140 | 11/15/2018 | 3/5/2020 |
| 2. Early-COVID | 59 | 3/6/2020 | 9/1/2020 |
| 3. Later-COVID | 107 | 9/2/2020 | 6/11/2021 |
| **30-day washout** |  |  |  |
| Excluded | 1 | 3/6/2020 | 3/6/2020 |
| 1. Pre-COVID | 140 | 11/15/2018 | 3/5/2020 |
| 2. Early-COVID | 68 | 4/30/2020 | 9/30/2020 |
| 3. Later-COVID | 97 | 10/6/2020 | 6/11/2021 |
| **60-day washout** |  |  |  |
| Excluded | 3 | 3/6/2020 | 5/1/2020 |
| 1. Pre-COVID | 140 | 11/15/2018 | 3/5/2020 |
| 2. Early-COVID | 81 | 5/5/2020 | 10/29/2020 |
| 3. Later-COVID | 82 | 11/2/2020 | 6/11/2021 |
| **90-day washout** |  |  |  |
| Excluded | 27 | 3/6/2020 | 6/2/2020 |
| 1. Pre-COVID | 140 | 11/15/2018 | 3/5/2020 |
| 2. Early-COVID | 68 | 6/5/2020 | 11/24/2020 |
| 3. Later-COVID | 71 | 12/8/2020 | 6/11/2021 |
| **120-day washout** |  |  |  |
| Excluded | 45 | 3/6/2020 | 7/1/2020 |
| 1. Pre-COVID | 140 | 11/15/2018 | 3/5/2020 |
| 2. Early-COVID | 60 | 7/7/2020 | 12/29/2020 |
| 3. Later-COVID | 61 | 1/4/2021 | 6/11/2021 |
| **150-day washout** |  |  |  |
| Excluded | 55 | 3/6/2020 | 7/30/2020 |
| 1. Pre-COVID | 140 | 11/15/2018 | 3/5/2020 |
| 2. Early-COVID | 58 | 8/20/2020 | 1/28/2021 |
| 3. Later-COVID | 53 | 2/3/2021 | 6/11/2021 |
| **180-day washout** |  |  |  |
| Excluded | 59 | 3/6/2020 | 9/1/2020 |
| 1. Pre-COVID | 140 | 11/15/2018 | 3/5/2020 |
| 2. Early-COVID | 64 | 9/2/2020 | 2/25/2021 |
| 3. Later-COVID | 43 | 3/1/2021 | 6/11/2021 |

**Supplementary Table 2B. Sensitivity Analyses for Differences in Office Visits between Time Periods**

|  |  | Pre-COVID vs. early COVID | | Pre-COVID vs. later COVID | | Early COVID vs. later COVID | |
| --- | --- | --- | --- | --- | --- | --- | --- |
| n | Washout periods | Event Ratio (95% CI) | *p* | Event Ratio (95% CI) | *p* | Event Ratio (95% CI) | *p* |
| 306 | 0 | 0.68 (95% CI: 0.47, 0.98) | 0.038 | 0.61 (95% CI: 0.45, 0.82) | 0.001 | 0.9 (95% CI: 0.61, 1.31) | 0.580 |
| 305 | 30 | 0.68 (95% CI: 0.48, 0.95) | 0.026 | 0.60 (95% CI: 0.45, 0.82) | 0.001 | 0.89 (95% CI: 0.62, 1.29) | 0.540 |
| 303 | 60 | 0.60 (95% CI: 0.43, 0.82) | 0.001 | 0.61 (95% CI: 0.44, 0.84) | 0.002 | 1.02 (95% CI: 0.71, 1.47) | 0.910 |
| 279 | 90 | 0.61 (95% CI: 0.43, 0.85) | 0.004 | 0.63 (95% CI: 0.45, 0.87) | 0.005 | 1.03 (95% CI: 0.7, 1.52) | 0.880 |
| 261 | 120 | 0.67 (95% CI: 0.47, 0.94) | 0.019 | 0.58 (95% CI: 0.41, 0.81) | 0.001 | 0.87 (95% CI: 0.58, 1.3) | 0.490 |
| 251 | 150 | 0.62 (95% CI: 0.44, 0.88) | 0.008 | 0.58 (95% CI: 0.41, 0.82) | 0.002 | 0.92 (95% CI: 0.6, 1.42) | 0.720 |
| 247 | 180 | 0.71 (95% CI: 0.51, 0.99) | 0.043 | 0.48 (95% CI: 0.33, 0.7) | 0.000 | 0.67 (95% CI: 0.44, 1.04) | 0.076 |

Note: CI = confidence interval. Adjusted negative binomial regression model controlled for age, education, poverty status, marital status, and disease stage. Data related to telephone and video visits were only collected after the COVID pandemic started, and thus comparisons to the pre-COVID period were not possible. Washout period included the number of days between pre- and early-COVID that were excluded from this sensitivity analyses to minimize overlap between pre- and COVID periods.

**Supplementary Table 2C. Sensitivity Analyses for Differences in Hospitalizations between Time Periods**

|  |  | Pre-COVID vs. early COVID | | Pre-COVID vs. later COVID | | Early COVID vs. later COVID | |
| --- | --- | --- | --- | --- | --- | --- | --- |
| n | Washout periods | Event Ratio (95% CI) | *p* | Event Ratio (95% CI) | *p* | Event Ratio (95% CI) | *p* |
| 306 | 0 | 0.95 (95% CI: 0.52, 1.73) | 0.870 | 0.81 (95% CI: 0.48, 1.34) | 0.410 | 0.85 (95% CI: 0.45, 1.61) | 0.610 |
| 305 | 30 | 1.10 (95% CI: 0.63, 1.91) | 0.740 | 0.70 (95% CI: 0.41, 1.2) | 0.200 | 0.64 (95% CI: 0.34, 1.19) | 0.160 |
| 303 | 60 | 1.19 (95% CI: 0.72, 1.97) | 0.500 | 0.57 (95% CI: 0.32, 1.04) | 0.069 | 0.48 (95% CI: 0.26, 0.92) | 0.026 |
| 279 | 90 | 1.10 (95% CI: 0.62, 1.94) | 0.750 | 0.55 (95% CI: 0.29, 1.06) | 0.074 | 0.50 (95% CI: 0.24, 1.04) | 0.064 |
| 261 | 120 | 1.27 (95% CI: 0.72, 2.24) | 0.410 | 0.52 (95% CI: 0.26, 1.04) | 0.066 | 0.41 (95% CI: 0.19, 0.89) | 0.024 |
| 251 | 150 | 1.02 (95% CI: 0.55, 1.88) | 0.960 | 0.55 (95% CI: 0.26, 1.17) | 0.120 | 0.54 (95% CI: 0.23, 1.27) | 0.160 |
| 247 | 180 | 1.06 (95% CI: 0.59, 1.91) | 0.840 | 0.48 (95% CI: 0.21, 1.13) | 0.094 | 0.45 (95% CI: 0.18, 1.14) | 0.093 |

Note: CI = confidence interval. Adjusted negative binomial regression model controlled for age, education, poverty status, marital status, and disease stage. Data related to telephone and video visits were only collected after the COVID pandemic started, and thus comparisons to the pre-COVID period were not possible. Washout period included the number of days between pre- and early-COVID that were excluded from this sensitivity analyses to minimize overlap between pre- and COVID periods.
